# The trinity of ecological contrasts: a case study on rich insect assemblages by means of species, functional and phylogenetic diversity measures

**DOI:** 10.1186/s12898-020-00298-3

**Published:** 2020-05-10

**Authors:** Elia Guariento, Patrick Strutzenberger, Christine Truxa, Konrad Fiedler

**Affiliations:** 1 Department of Botany & Biodiversity Research, University of Vienna, Rennweg 14, 1030 Vienna, Austria; 2 Present Address: Institute for Alpine Environment, Eurac Research, Via Druso 1, 39100 Bolzano/Bozen, Italy

**Keywords:** Near-annual inundations, Moths, Riparian forest, Diversity metrics, Community pattern analysis

## Abstract

**Background:**

The ‘classical’ concept of species diversity was extended in the last decades into other dimensions focusing on the functional and phylogenetic diversity of communities. These measures are often argued to allow a deeper understanding of the mechanisms shaping community assembly along environmental gradients. Because of practical impediments, thus far only very few studies evaluated the performance of these diversity measures on large empirical data sets. Here, data on species-rich riparian moth communities under different flood regimes and from three different rivers has been used to compare the power of various diversity measures to uncover ecological contrasts.

**Results:**

Contrary to the expectation, classical metrics of species diversity (Hill numbers N1, N2 and N_inf_) and evenness (Buzas-Gibson’s E and Pielous’s J) turned out to be the most powerful measures in unravelling the two gradients investigated in this study (e.g. flood regime and region). Several measures of functional and phylogenetic diversity tended to depict either only one or none of these contrasts. Rao’s Q behaved similarly as species diversity and evenness. NTI and NRI showed a similar pattern among each other but, were different to all the other measures. Functional Divergence also behaved idiosyncratically across the 28 moth communities. The community weighted means of nearly all individual functional traits showed significant ecological patterns, supporting the relevance of the selected traits in shaping assemblage compositions.

**Conclusions:**

Species diversity and evenness measures turned out to be the most powerful metrics and clearly reflected both investigated environmental contrasts. This poses the question when it is useful to compile the additional data necessary for the calculation of additional diversity measures, since assembling trait bases and community phylogenies often requires a high work load. Apart from these methodological issues, most of the diversity measures related to communities of terrestrial insects like moths increased in forests that still are subject to flooding dynamics. This emphasizes the high conservation value of riparian forests and the importance of keeping and restoring river dynamics as a means of fostering also terrestrial biodiversity in floodplain areas.

## Background

Throughout the 20th century, biodiversity at the community level has largely been studied using species as the primary units of analysis. Studying the numbers and relative abundances of species continues to form the backbone of much current biodiversity research [1]. During the past decades, however, two important extensions have been developed: the concepts of functional [2] and phylogenetic diversity [3], termed FD and PD hereafter. FD concentrates on the frequency distributions of functional traits, rather than mere species identities. This approach aims at a more mechanistic understanding of differences in biodiversity along environmental gradients [4]. Various studies have demonstrated the usefulness of FD approaches which sometimes appeared superior to species-based analyses in unravelling ecological patterns and processes [5, 6]. PD, on the other hand, addresses the phylogenetic similarity (or dissimilarity) amongst organisms within communities. The underlying paradigm here is that many traits which determine the occurrence of species in ecological niche space have a genetic basis that is inherited from phylogenetic ancestors during evolution and speciation. As a corollary, processes such as environmental filtering ([7], refined by Cadotte and Tucker, [8]) or limiting similarity [9] are expected to leave a phylogenetic signature in the composition of local communities that are assembled from regional species pools. An inherent assumption in the interpretation of PD is that the more closely related organisms are, the more traits they share by descent and therefore tend to occupy more similar niches than is the case with distantly related organisms (i.e. the principle of phylogenetic conservatism [10]). Along those lines, Faith [11] introduced the term ‘feature diversity’ encompassing morphological and functional diversity [12]. This proposed surrogacy of PD for FD has frequently been invoked as a paradigm in conservation biology [13, 14] despite the lack of empirical evidence [15].

For the quantitative analysis of FD as well as PD, a wide range of mathematical measures have been developed, often based on extensions of concepts that are already well established in traditional species diversity (SD) research, such as Shannon’s entropy [16]. While the conceptual advantages of FD and PD approaches over mere ‘species counting’ are obvious from their theoretical foundations (especially in niche theory), both these concepts are confronted with severe impediments in practical research. For many organisms—especially for arthropods—the sequence information required to calculate meaningful PD measures is either unavailable or of limited use due to the coexistence of several provisional taxonomic systems. Furthermore, the reconstruction of well-resolved phylogenetic trees relies on the availability of published phylogenies for the taxon in question. Collating trait matrices required for analyses of FD is even more challenging [15]. It is essential to assemble a trait matrix which, in many cases, must be derived from databases or a large array of literature. This procedure is obviously contingent on proper species identifications of samples and therefore requires taxonomic expertise for the focal groups in question. More importantly, ecologists also face the challenge of lacking data for many organisms, especially from under-explored geographical regions or taxa.

Those heavy impediments acting on studies working with PD and FD resulted in a remarkable lack of literature comparing approaches based solely on species identities with those based on functional traits, or on phylogenetic relatedness, applied to the same real-world community samples. It is therefore difficult to assess if, and how strongly, the outcome of biodiversity analyses along environmental contrasts might differ between these approaches. Most available evidence comes from large scale studies. North American tetrapod vertebrates [17] showed a high degree of correspondence between SD, FD and PD while global analyses of birds, mammals and reef fishes [18] revealed an overall poor correspondence between FD and PD with considerable geographic and among taxon variation.

We here use a large data set on a species-rich group of terrestrial insects (448 species; > 32,000 individuals identified to species level) to compare the performance of SD, FD and PD measures in revealing patterns along two well defined ecological contrasts. As a target group, we have chosen nocturnal moths. Taxonomy of moths in Central Europe is well established, and thanks to 250 years of recording by naturalists, a wide variety of traits is documented for almost all species. Moreover, intense campaigns during the past 15 years to assemble DNA barcode libraries (using a part of the mitochondrial CO1 gene [19]) resulted in a near-complete coverage of Central European moth species [20, 21], enabling us to use this data to reconstruct species-level phylogenies based on published backbone phylogenies. Therefore, moths are probably nearly unique among species-rich European insect clades with regard to the simultaneous coverage of traits as well as available DNA barcodes. Finally, by use of light-trapping, large representative samples of moths can be collated with manageable work load [22]. The samples explored here for their FD and PD patterns have previously been shown to precisely mirror ecological contrasts between three riverine regions and two flood regimes in conventional species-level analyses [23, 24].

Against this background, we addressed the following research questions:

1. Do individual functional traits capture the signature of the flood regimes?
2. Do multivariate FD measures track compositional differences in the moth assemblages between regions and especially between flood regimes?
3. Are PD measures able to reveal these two interacting sets of ecological contrasts?

Flood regimes are known to act as important environmental filters on terrestrial arthropods. Recurrent inundations reduce FD (and similarly also PD) and select for traits associated with higher mobility and faster re-colonization [4, 25]). Among Lepidopterans, especially the less mobile early stages (eggs, larvae, and pupae) experience inundation of their habitats as a major cause of mortality [23, 26]. We therefore expected that moth assemblages in forest stands subject to near-annual floods should be constrained by this natural disturbance. As a consequence, species should become relatively more prevalent in flood-prone forests that share traits which lower their mortality risks during inundations and/or which enable them to more quickly re-colonize such habitats after flood events.

## Results

**Fig. 1:**
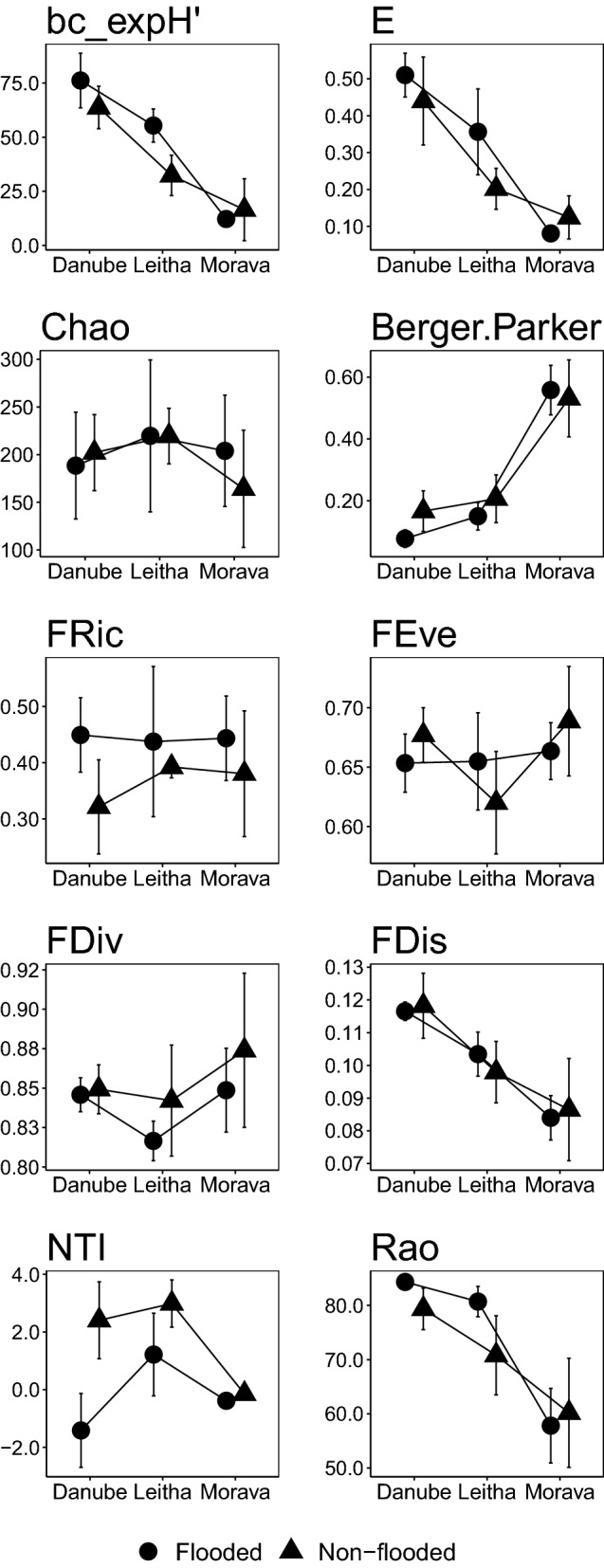
Means (95% confidence limits) of ten measures for species, functional and phylogenetic diversity across 28 moth assemblages in three east Austrian riverine regions, according to the flood regime of the forest stands. Panels refer to: bias-corrected exponential Hʹ (bc_expHʹ); Buzas-Gibson’s evenness (E); extrapolated species richness (Chao); species dominance (Berger.Parker); functional richness (FRic); functional evenness (FEve); functional divergence (FDiv); functional dispersion (FDis), nearest taxon index (NTI); and Rao’s Q metric of phylogenetic diversity

Moth species diversity (SD) was highest in the forests at river Danube, lowest at Morava, and intermediate at river Leitha (Fig. 1). This sequence was equally strongly reflected by the Hill numbers N1 (exponential Shannon Hʹ, with or without bias correction), N2, and N_inf_, Menhinick’s diversity metric, as well as by evenness (in Pielou’s or Buzas and Gibson’s variant). In both the Danube and Leitha regions, moth species diversity and evenness was distinctly higher in flood-prone as opposed to non-flooded forest stands, while no significant variation in local species diversity was observed among the Morava samples (Fig. 1, Table 1). Observed or extrapolated species richness were completely uninformative, as was Margalef’s diversity index. Other species diversity metrics only mirrored differences between the three regions, but showed no signature of flood regimes, such as Fisher’s alpha, Berger-Parker’s dominance, Simpson’s lambda, the logarithmic version of Shannon’s Hʹ, or Brillouin’s diversity index (Table 1; see also Additional file 1).

**Table 1:**
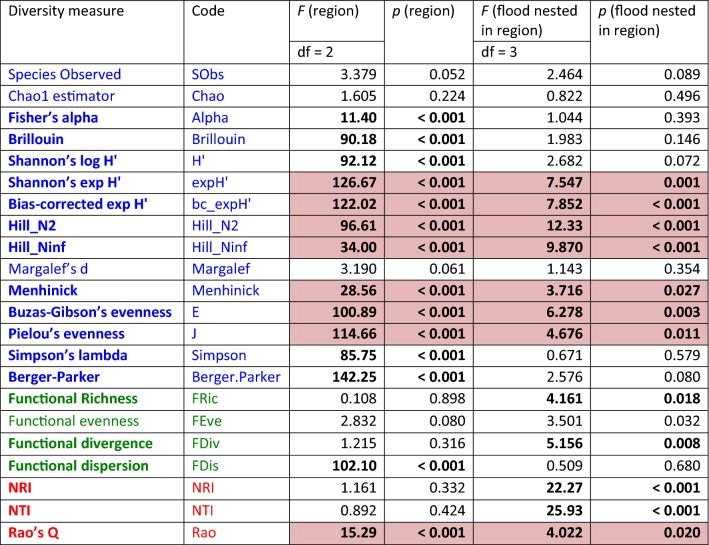
Statistical significance of differences between three riverine regions and two flood regimes with regard to various measures of species diversity (blue), functional diversity (green) and phylogenetic diversity (red)

Results of nested ANOVAs (*F* and *p* values). Statistically significant results (corrected for a table-wide false discovery rate at *p *< 0.05: [27]) are printed in bold face. Diversity measures that revealed significant effects of both, region and flood regime, are shaded in colour

**Fig. 2:**
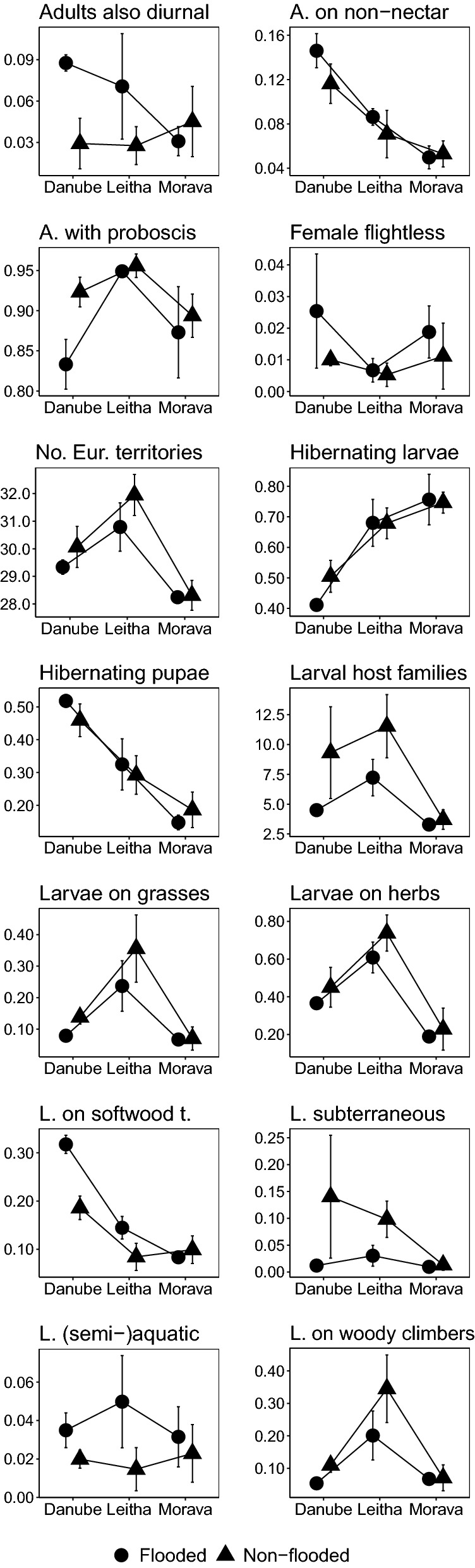
Community weighted means (95% confidence intervals) of the 15 moth species traits that showed significant effects of the flood regime, across the three riverine forest regions in easternmost Austria

Except for one single trait (caterpillars gregarious: yes or no?), the remaining 26 species traits all turned out to be ecologically informative in the environmental gradients under scrutiny. In ANOVA comparisons community-weighted means (CWMs) of 13 traits revealed significant differences between the three regions and the two flood regimes, 11 traits revealed only regional differences, while two traits differed only in regards to the two flood regimes (Table 2). This indicates non-random community shifts among moths related to near-annual inundation events. Specifically, moths in flood-prone areas tended to have smaller distributional ranges in Europe; included more species with partially diurnal adult activity (not at Morava); had a higher incidence of species that utilize non-nectar resources (such as rotting fruits) during the adult stage (again not at Morava); more frequently have a reduced non-functional proboscis (i.e. comprise a higher fraction of capital breeders, especially at Danube); have larvae with more narrow host-plant ranges; are less likely to have subterraneous larvae (not at Morava); comprise more herbivores on softwood trees such as poplars and willows (not at Morava); include fewer herbivores on woody climbers such as *Clematis vitalba* and *Hedera helix* (not at Morava); more frequently have larvae feeding on reed or submerse aquatic plants; and finally comprise fewer herbivores of herbs as well as of grasses (Fig. 2).

**Table 2:**
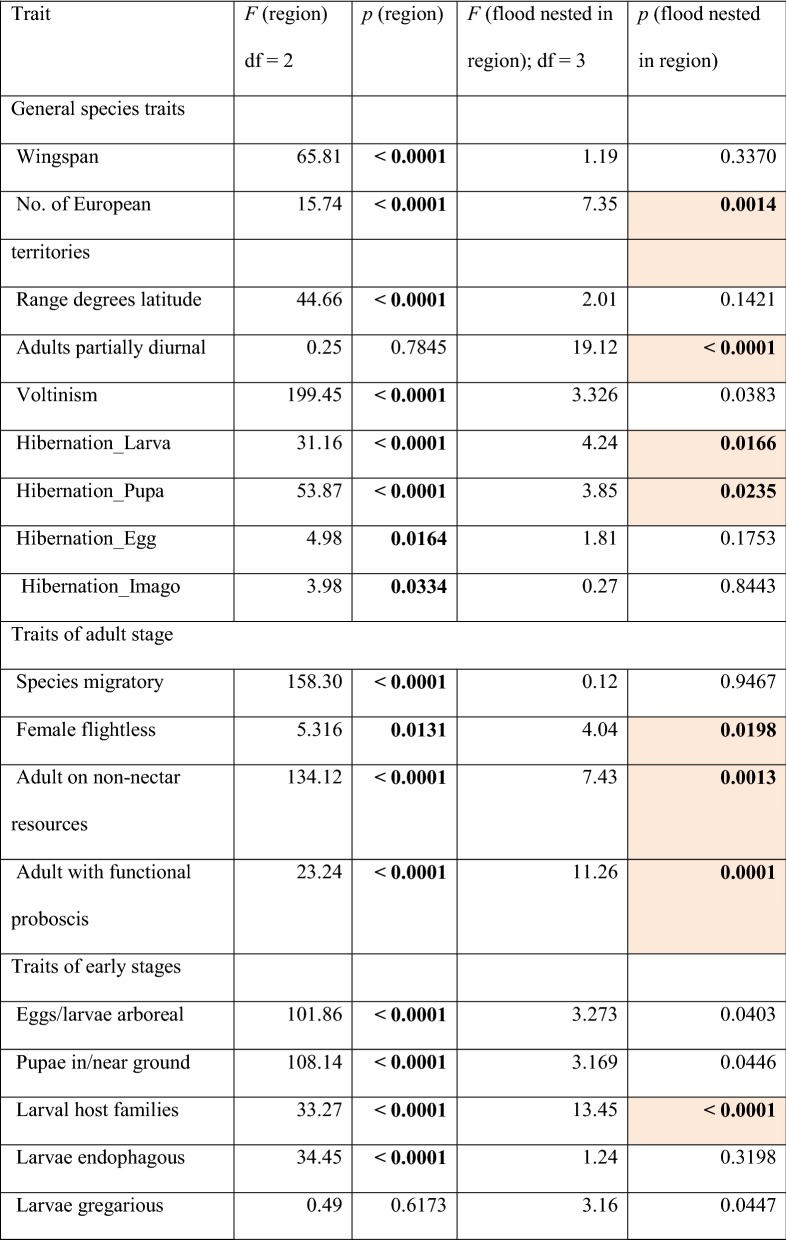

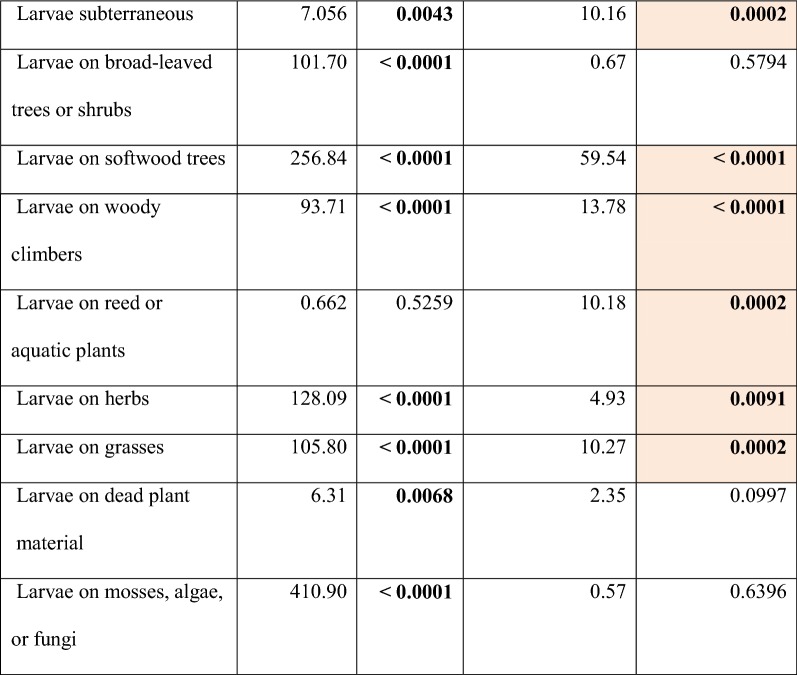
Statistical significance of differences between three regions and two flood regimes with regard to community-weighted means (CWMs) of 27 traits of moth species

Results of nested ANOVAs (*F* and *p* values). Statistically significant results (corrected for a table-wide false discovery rate at *p *< 0.05: [27]) are printed in bold face. Traits with significant difference according to flood regime are shaded in colour

The four multivariate metrics of functional diversity (Table 1) revealed quite variable results (Fig. 1). Functional richness (FRic) showed a completely different pattern across sampling sites than all measures of species diversity, with no significant variation between the three riverine regions, but a consistent increase in flood-prone forest stands relative to those with little or no inundation impact. In contrast, patterns were inconsistent with regard to functional evenness (FEve): at Danube and Morava, FEve was slightly lower at flood-prone than non-flooded sites, but the reverse was true for the Leitha sites. No differences were seen between the three regions in the overall (and rather low) level of FEve. Functional divergence (FDiv) of moth assemblages was consistently lower in flood-prone forest stands than in non-flooded ones (also at Morava), whereas again no general differences emerged between the three study regions. Finally, functional dispersion (FDis) showed a clear segregation between the regions analogous to species diversity and evenness, with values at Danube being highest, lowest at Morava, and intermediate in the forests at river Leitha. However, flood regime did not leave a detectable signature in FDis.

Among the measures of phylogenetic diversity, abundance-weighted NRI and NTI showed very similar patterns: there were no significant differences between the three riverine regions, but both measures attained consistently lower values in flood-prone forest stands (Fig. 1). These differences were particularly pronounced at the rivers Danube and Leitha, where also the distinction between flood-prone and non-flooded forest stands, separated by levees, was stronger than at the river Morava. The pattern observed with Rao’s Q (Fig. 2) paralleled the results for species diversity and evenness, arriving at the ranking of Danube > Leitha > Morava between the three regions. Rao’s Q increased towards flood-prone sites for the moth assemblages at Danube and Leitha, whereas no clear difference according to flood status was observed in samples taken at river Morava.

**Fig. 3:**
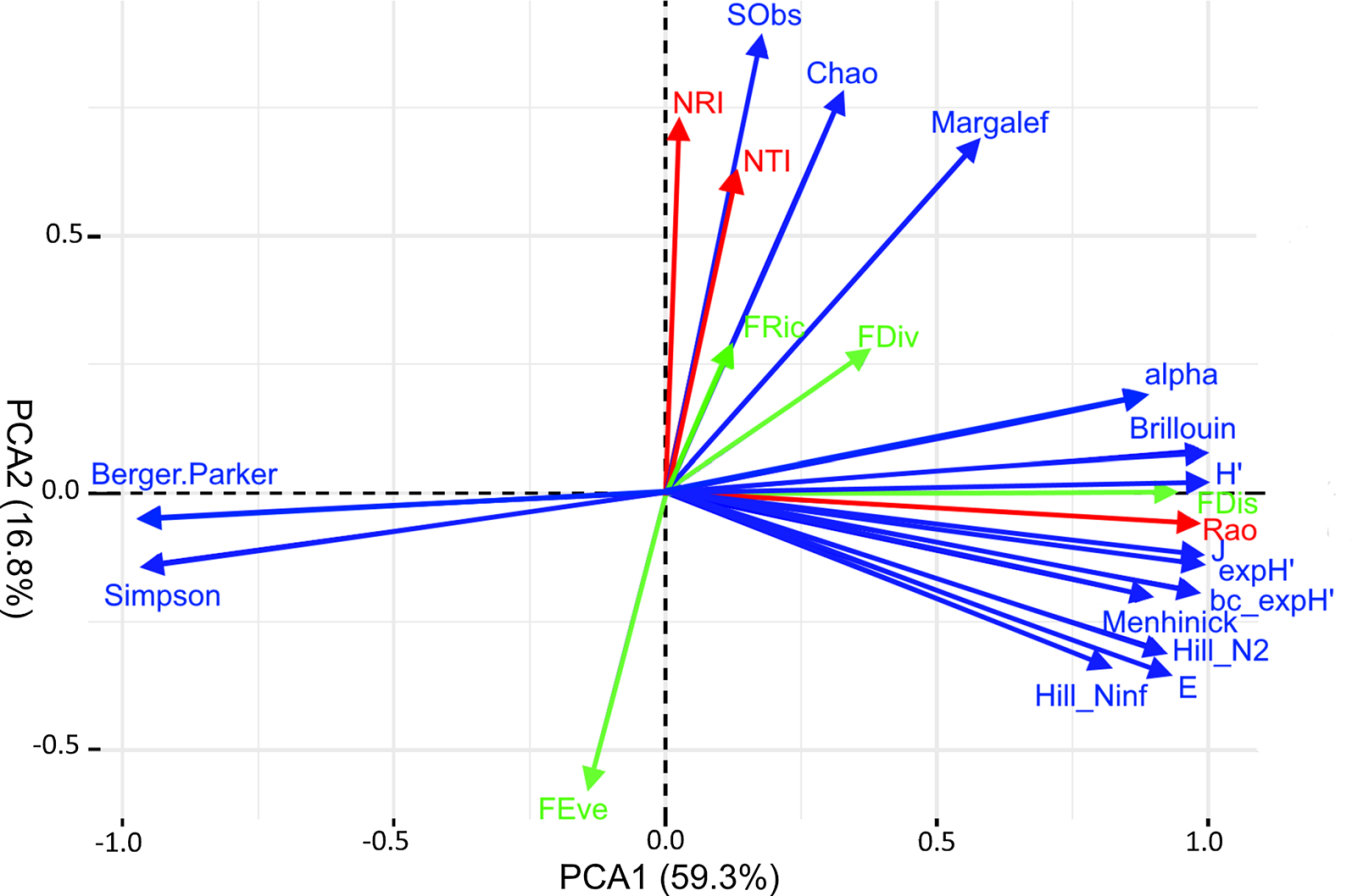
Principal components ordination of 22 community diversity metrics (as vectors), compared across 28 moth communities in East Austrian floodplain forests. The first two ordination axes together account for 76.1% of variation in the data. SD and evenness metrics in blue, FD measures in green, and PD measures in red

Overall, metrics of SD, FD and PD revealed surprisingly little concordance across the 28 moth assemblages under study. Strong positive correlations were only seen between various measures of species diversity, evenness, Rao’s Q and FDis (see Additional file 2). Moreover, as expected, NTI and NRI were substantially related to each other. Otherwise, the various community diversity metrics varied in a largely idiosyncratic manner (*r*-values < 0.500). A PCA of the 22 community metrics (Fig. 3) revealed that (a) most metrics of species diversity and evenness, FDis and Rao’s Q measure largely shared the same information content with regard to the moth assemblages at the 28 sites, spanning along the first PC axis; (b) NTI, NRI, observed and extrapolated species richness formed a second group; (c) FEve formed a third case, essentially antiparallel to NTI and NRI in reduced ordination space; and (d) FDiv and Margalef’s index were widely unrelated to any of the other community diversity metrics.

## Discussion

### Inundation and moth diversity

The species rich moth communities in the three riverine regions of easternmost Austria revealed a strong patterning along environmental contrasts which is congruent with many terrestrial ecosystems all over the world [28–31]. In particular, flood regime left a clear signature in 12 out of 22 diversity metrics under consideration. SD and evenness tended to be higher in forest stands that experience near annual inundations, as opposed to those cut off from river dynamics by levees. This can be attributed to enhancement of diversity through disturbance events that create novel niches or prevent competitively superior species from outcompeting others, as long as these disturbance episodes are not too severe or do not occur too frequently. A similar pattern in species diversity has been observed with wild bees inhabiting meadows in one of the riverine regions studied herein for forest moths, viz. along the Danube. There, species richness was higher on flood-prone meadows than on meadows behind the levee [32]. For meadow butterflies, on the other hand, a moderate reduction in species diversity was observed in relation to inundation risk in the same region [33].

In line with the above interpretation, FRic of moth assemblages was consistently higher in flood-prone forest stands than in those protected from inundations by levees. In contrast, FDiv was slightly, but routinely lower in flood-prone forest stands. This indicates that moth species which make up local communities under regular inundation impact occupy a smaller fraction of the trait space relative to the entire regional species pool. This observation precisely mirrors the expectation under the concept of environmental filtering, i.e. only a fraction of somehow ‘flood-adapted’ species is able to attain higher population densities in forest stands with inundation risk.

The analysis of individual species traits broadly confirms this conclusion. In most cases where statistically significant differences in the CWMs could be found in relation to the flood regime, these concur with what one would have expected from the outset. For example, subterraneous larvae or herb and grass feeders were less common in inundated forest stands. This perfectly matched the higher mortality risks of these life styles during flood episodes. Conversely, species whose larvae feed on wetland plants like softwood trees, reed, or on water plants were more prevalent in flood-prone forest stands. Moth species in inundated habitats also had, on average, narrower larval host plant ranges. This reflects the vegetation conditions in the forests under study, where in flooded sites just a few plant species tended to dominate the herb as well as tree layers. Woody climbers such as *Clematis vitalba* and *Hedera helix* occurred far more frequently in the non-flooded forest parts, and accordingly herbivores of these plants were more prevalent there. Overall, the consideration of individual species traits corroborates the preponderance of non-random distribution of moths along the flood-impact axis and therefore yields ample support for the concept of environmental filtering to be important in community assembly.

PD metrics of moth communities showed two different patterns. On the one hand, Rao’s Q (which was overall highly correlated with SD and evenness across the 28 assemblages) revealed a minor, but significant increase in diversity at least in the two riverine regions where the forests representing the two flood regimes were separated from the respective river by a levee (viz. Danube and Leitha). On the other hand, NTI and NRI indicated that in flood-prone forest stands moth assemblages were phylogenetically less clustered than in forest areas with smaller or no inundation impact. Apparently, inundation therefore reduces phylogenetic clustering. A possible explanation for this somewhat unexpected observation is that local extinctions or temporary population declines during inundation episodes result in communities further away from equilibrium, which are then more strongly shaped by random processes as opposed to the ‘climax communities’ in forest stands without flood disturbance. Our observation also indicates that there are, in our study region, likely no larger moth clades that are per se better, or worse, adapted to surviving floods. Hence, flooding does not lead to stronger phylogenetic clumping, whereas for example along elevational gradients an increase in phylogenetic clustering has been found in moths [34] and many other organisms (e.g. ants: [35]; birds: [36, 37]). As argued by Cadotte and Tucker [8], environmental filtering need not be mediated solely by abiotic interactions but can represent abiotic and biotic interactions acting in combination. The patterns observed here might also be shaped by competition, predation and even more complex, possibly bidirectional, interactions with other organisms. Separating those effects from environmental (= abiotic) filtering in the strict sense [38] is not possible with the available data. It is conceivable that inundation regimes modify annual vegetation phenology in a way that favours certain species while putting others at a disadvantage, mediated by changes in synchronization of host plant phenology with moth life histories. Several studies found evidence that changes in plant phenology can be disruptive to herbivore populations [39–41].

### Regional differences between moth assemblages

Species diversity and evenness of moth assemblages differed drastically between the three riverine regions, as already demonstrated earlier [24]. This was mainly due to the massive dominance of a few common species in the forests at river Morava (as shown by the Berger-Parker index), which reduced the numerical values of all other conventional SD and evenness indexes. We observed the same regional pattern with FDis and Rao’s Q, but only Rao’s Q simultaneously captured any flood effect. Dispersion of moth species in trait space was obviously the lowest in the forests at river Morava, which again reflects the hyper-dominance of a few species there. Otherwise, neither the FD nor the PD measures captured differences between the three riverine regions. This means that there were no consistent changes in the extent of phylogenetic clumping between forest moth assemblages at Danube, Leitha and Morava, and also with regard to the breadth of occupied trait space all these moth communities were essentially similar.

We did not have any a priori expectations with regard to a regional ranking of moth assemblages based on functional traits or phylogenetic relatedness of component species. Hence, the widespread absence of such patterns was not surprising. Yet, given the strong gradient in species diversity and evenness, it is remarkable that in this regard most FD or PD metrics were not concordant to the analysis based on SD, whereas other indexes of species diversity, such as rarefied species richness or Fisher’s alpha index, well captured the same gradient, with moth assemblages at river Danube attaining highest, and those at Morava the lowest, values [24].

However, as with inundations also the three regional affiliations of the study sites revealed strong signatures on the level of individual species traits. Only three traits were not informative along this geographic gradient: the fraction of species with partial diurnal activity during the adult stage, the incidence of larval gregariousness, and the incidence of species with (semi-)aquatic larval stages were roughly equal in all three regions. Otherwise, trajectories of individual traits varied widely across regions. The most frequently observed pattern was a maximum CWM value for the samples taken at river Leitha, but a rank order of Danube > Leitha > Morava (or the reverse) also occurred repeatedly. These patterns likely mirror differences in the vegetation composition and thus resource availability for various moth guilds, but specific data on vegetation features would be required to more rigorously assess such associations.

### Comparative performance of SD, FD and PD metrics

The various metrics of SD, FD and PD assessed in the present study revealed only limited co-variance. This indicates that indeed the concepts underlying these measures capture quite different aspects of ‘biodiversity’ and therefore they may often merit proper analysis in their own right. Yet, FDis and Rao’s Q showed very close relationships with multiple SD and evenness metrics and therefore turned out to be redundant to the latter in our case study. Since FDis failed to capture the differences in composition of moth assemblages between flooded and non-flooded forest stands, this latter measure can be regarded as having too low power here, even though trait data could be assembled in an unusually complete manner. It is also worth emphasizing that neither observed nor extrapolated species richness were informative with regard to the regional differentiation or flood regimes in our case [24]. We attribute this failure to the large fraction of singletons or otherwise ‘rare’ species in samples of insects as mobile as moths are. Similarly, the ‘classical’ logarithmic version of Shannons’s Hʹ as well as the mathematically related Brillouin index failed in uncovering the impact of flood regime on moth community structure.

Of the three PD measures, NTI and NRI were also closely related to each other in their pattern across the 28 study sites, but completely unrelated to most SD and evenness metrics, as well as Rao’s Q and FDis. Hence, the mean relatedness of moth species within communities addresses a perspective of diversity that is clearly distinct from species diversity or functional dispersion. Remarkably, NTI and NRI turned out to be the most sensitive metric with regard to inundation effects on moth assemblages. Especially at the rivers Danube and Leitha, moth ensembles in forest stands that are decoupled from riverine dynamics by levees since many decades were phylogenetically more clustered than in the flood-prone stands. This might indicate that due to the lack of recurrent disturbance events related to inundations, in these drier forest stands communities have developed towards a stronger influence of resource partitioning and competition. This is what one might expect in ecosystems that are in a later stage of succession, on the trajectory towards a new type of climax vegetation.

Two FD measures, viz. FEve and to a much lesser extent FRic, were correlated with the two PD metrics NTI and NRI and therefore also emerged as somewhat redundant, but less informative (lower correlation coefficients, shorter vectors in reduced ordination space) than the latter two. Finally, the fourth FD metric, FDiv, showed a largely idiosyncratic behaviour. Values were weakly and non-significantly related to NTI and NRI. But like the latter FDiv was also sensitive to the flood regime, attaining lower values in flood-prone forest stands. This is consistent with the expectation that near-annual flood events restrain the fraction of niche space to be occupied by terrestrial insects.

## Conclusions

Overall, none of the tested FD and PD measures was superior to ‘conventional’ analysis based on SD or evenness metrics in simultaneously detecting signatures of the two ecological gradients (regions, flood regimes) in the species-rich insect assemblages under study. Only Rao’s Q performed similar to species diversity measures from the Hill series such as N1, N2 or N_inf_, as well as to the evenness metrics E and J. However, the inundation effect was just weakly visible with Rao’s Q. Therefore, our case study does not support the view that trait-based analyses were per se more informative than species-based approaches. Yet, we corroborated that at least some of these diversity metrics elucidate complementary aspects of community patterns along gradients and therefore do merit to be assessed in parallel. For example, NTI and NRI captured a far stronger signal of inundation influences on moth assemblages than any other SD, FD or PD index. It should also be noted that assembling a large trait matrix with dozens of traits and hundreds of species will often meet severe limitations through data availability. For few species-rich insect groups, even in Central Europe, will it be feasible to locate the information needed in literature and data bases. Hence, the extra workload (in addition to species identification) will not always be rewarded by higher ecological resolution, a trade-off that is crucial to consider in biodiversity monitoring [42].

On the other hand, addressing multiple individual species traits enables a far better mechanistic understanding of the processes during community assembly that shape the differences in species composition. This was also clearly the case with the hundreds of moth species in our study, where CWMs were particularly informative with regard to the strength and direction of inundation effects.

Finally, our analyses confirm that cutting off floodplain forests from the hydrological dynamics of their rivers has profound repercussions also on rich fractions of terrestrial fauna. Not only was species diversity, evenness and functional richness of moth assemblages higher in flood-prone forest stands, but also their phylogenetic clustering was decidedly lower. These findings underline how important it is to preserve and restore riverine dynamics in an attempt to conserve biodiversity of these threatened and declining habitats.

## Methods

### Study sites

Moths were collected using light-traps at 28 sites situated in lowland forest stands along the three rivers Danube, Morava and Leitha in easternmost Austria. In each of these regions, half of the trap sites were located in forest stands that receive inundations almost every year (termed ‘flood-prone’ hereafter), whereas the other trap sites (‘non-flooded’) were selected in stands that are cut off from flood dynamics by levees since decades (at the rivers Leitha and Danube) or are affected only for distinctly shorter times per year by inundations (Morava). Hydrodynamic conditions vary between the three rivers [43]. While at the Danube, floods mostly occur in summer when alpine snow-melt coincides with spells of heavy rain, inundations are more concentrated in spring at the other two rivers and typically follow periods of intense rainfall. During the period of our moth sampling (years 2006–2008), ‘flooded’ forest stands were altogether inundated for approximately 40 days (Danube), 190 days (Leitha), and 110 days (Morava), respectively. Also, the forest vegetation along the three rivers shows characteristic differentiation. See [43] for a detailed description of the study regions and light-trap sites.

### Field work

Moths were collected using automated light traps (source: http://www.fiebig-lehrmittel.de), equipped with two 15 W tubes (Sylvania Blacklight-Blue, F15W/BLB-T8; and Philips TLD, 15W/05) and powered by a 12 V car battery. These weak lamps essentially sample moths only from their immediate surroundings [44]. At dusk the light was automatically switched on and run for about 6 h. All 4–5 light traps situated within one forest stand (trap sites being separated by at least 100 m from another) were operated simultaneously. All six forest areas were sampled on consecutive days, or as soon as possible if spells of unfavourable weather had to be avoided. We did not run traps during the 5 days before and after full moon to circumvent the impact of moonlight on trap catches [45]. Sampling did also not occur during rainy weather, in which cases light-trapping was postponed until weather conditions improved again.

Traps were placed about 1 m above ground under a closed forest canopy and run once a month during the vegetation period. Altogether, sampling went over two complete annual cycles on 103 nights between 20.VIII.2006 and 24.VIII.2008. The sampling season ended with the first incidence of frost in autumn (latest sampling dates: 26.XI.2006 and 08.XI.2007, respectively) and started again in spring (earliest sampling dates: 26.III.2007 and 07.IV.2008, respectively). We concentrated on the ‘macro-moths’ (essentially the representatives of’eared moths’ sensu [46] including the Pyraloidea, plus single species of Hepialidae, Cossidae, and Limacodidae), which we identified to species level using faunal monographs, if necessary also using genitalic dissections. An additional spreadsheet file enlists all recorded species per site (see Additional file 3).

### Trait data

For all 448 moth species recorded in light-traps, we assembled a wide range of 27 traits that describe various aspects of the larval and adult ecological niche occupied, including body size, geographic range size, resource use during adult and larval stages, voltinism, hibernation stage, and micro-habitat use. Two additional spreadsheet files contain a full list of the traits per species (see Additional file 4) and their scaling and the sources used to assemble the trait matrix (see Additional file 5).

### Phylogenetic reconstruction

We queried the Barcode of Life Datasystems (BOLD) [47] for all species in the community data matrix of [43] through the BOLD Public Data Portal. From the obtained search results, we selected one COI barcode sequence per species with a minimum length of 600 bp. After nomenclatorial discrepancies were resolved we were able to obtain suitable sequences for 441 out of 448 species (~ 98%), see the additional spreadsheet for more details (see Additional file 6). The sequence dataset can be accessed directly at: http://dx.doi.org/10.5883/DS-FLOOD. In three cases we substituted a closely related species for species with no available DNA barcode.

Tree reconstruction was performed in a two-step approach. Step one involved using DNA barcodes along with a backbone constraint to estimate the tree topology. In step two, time calibrated node ages were estimated onto the tree topology obtained in step one. All species in our community sample with available DNA barcodes were grafted onto the consensus tree from [48] as basal polytomies on family level. Tree topology was estimated with RAxML version 8.2.11 [49]. Data was partitioned according to codon positions as recommended by results from Partitionfinder v1.1.1 [50] and a GTRGAMMA model was applied to all three partitions. The constraint tree was passed to RAxML using the -g option. Calculations were performed with the -f a option performing 25 runs to estimate the best-known-likelihood tree (BKL).

In step two we used BEAST v2.5.1 [51] to obtain a time calibrated ultrametric tree. The minimum age of all families, the split between Geometroidea and Noctuoidea, and the root were calibrated with ages obtained from [52]. Following results from Partitionfinder we applied a partitioning scheme with one partition per codon position and the GTR + I + G applied to the 2nd and 3rd codon position, and a GTR + G substitution model applied to the 1st codon position. Trees were estimated with a single log-normal-relaxed clock model and a Yule tree prior. The mcmc chain was run for 11 million generations, sampling every 1000th generation, resulting in a tree sample comprising 11,000 trees. Tree topology was constrained to the topology obtained from RAxML by setting the weights of the ‘narrow exchange’, ‘wide exchange’, ‘subtree slide’, and ‘wilsonbalding’ operators to zero. Calculations with BEAST were performed on the CIPRES Science Gateway [53] using the BEAGLE 2.1 library [54]. The resulting tree sample was examined with Tracer v1.7 to assess convergence of BEAST analyses and ensure sufficient effective sample sizes for all parameters (> 200). TreeAnnotator v2.5.1 was used to summarize the BEAST tree sample, common ancestor node heights were annotated to the maximum clade credibility tree. The first 1000 trees were removed from the sample as burn-in.

### Data analysis

We calculated the bias-corrected version of the exponential Shannon index bc_expHʹ using the package ‘SpadeR’ [55]. In a previous analysis of the same data [24] this alpha-diversity measure emerged as the most ecologically powerful one. In addition, we explored a wide range of community metrics that are conventionally used to express species diversity, evenness, or dominance in ecological communities. These included the logarithmic version of Shannon’s Hʹ, Brillouin’s diversity, Fisher’s alpha, Simpson’s lambda, the Hill numbers N1, N2 and N_inf_, Pielou’s as well as Buzas and Gibson’s evenness, Berger-Parker’s dominance, and the indexes suggested by Menihinck and Margalef [62]. Community weighted means (CWM) of each functional trait were calculated in the package ‘FD’ [56], as were multivariate functional diversity measures combining the trait matrix with the species-abundance matrix. For the latter, analysis was based on a Gower distance matrix of the traits implementing a Cailliez correction to account for negative eigenvalues. Traits partitioned in different columns (such as hibernation stage, see Additional file 4: Table S1) were weighted as one single trait in multivariate analysis. Phylogenetic diversity metrics were calculated using the R packages ‘pez’ [57] and ‘picante’ [58]. Input data were the tree obtained from BEAST and the community matrix from [43]. NRI and NTI [59] were calculated under the “phylogeny.pool” null model performing 10,000 iterations with abundance-weighting enabled. Rao’s Q [60] was calculated using default settings. Additional data handling in R was performed using the ‘ape’ package [61]. Subsequently, all community diversity measures were tested for their response to the ecological contrasts amongst the study sites using two-way ANOVAs, with the factor flood regime nested in regions. Furthermore, we explored co-variance between the various community diversity metrics across the 28 moth samples using a conventional principal components analysis (PCA) in the PRIMER v7 software [62].

## Supplementary information

**Additional file 1.** Overview of supplementary measures of species diversity, evenness and phylogenetic diversity of moth communities. Means (95% confidence limits) of 12 additional measures for species and phylogenetic diversity across 28 moth assemblages in three east Austrian riverine regions, according to the flood regime of the forest stands. Panels refer to: observed specie richness (Sobs); Pielou’s evenness (J); Simpson’s lambda; Fisher’s alpha; Shannon’s logarithmic diversity (Hʹ); Shannon’s exponential diversity (expHʹ); the indexes of Margalef, Menihinck, and Brillouin; the Hill numbers N2 and N_inf_; and the net relatedness index (NRI).

**Additional file 2.** Correlations between 22 measures of species diversity (and evenness), functional diversity, and phylogenetic diversity. Graphical display of strength and direction of Pearson correlations between 22 measures of species, functional, and phylogenetic diversity across 28 moth assemblages. Blue: positive correlations; red: negative correlations; shaded in grey: statistically significant after sequential Bonferroni correction. More narrow and darker ellipses indicate stronger covariance.

**Additional file 3.** Species × site matrix of moths sampled in automated light-traps at 28 floodplain forest sites in 3 riverine landscapes in easternmost Austria. Figures in the table cells denote the numbers of specimens per each species and site. Letters D, L and M in the site codes refer to the rivers Danube (D), Leitha (L), and Morava (M), respectively; letters N and F in the site codes refer to non-flooded (N) or annually flood-prone (F) forest stands. Further detailed information on the light-trap sites can be found in the PhD thesis of C. Truxa (http://othes.univie.ac.at/25605/).

**Additional file 4.** List of values for 27 species traits for 447 moth species sampled in automated light-traps at 28 floodplain forest sites in 3 riverine landscapes in easternmost Austria. For the sources extracted and the definition and scoring of the traits, see Additional file 5.

**Additional file 5.** Additional information concerning the origin and scoring of traits enlisted in Additional file 4. Sources for species trait information on 447 moth species sampled at 28 forest sites in 3 riverine regions of lowland easternmost Austria, and a list of the traits and their scoring for analysis.

**Additional file 6.** List of sequences of the barcoding region of the mitochondrial COI gene of 441 moth species sampled in 3 riverine landscapes in easternmost Austria. Data used to generate the phylogenetic tree for calculation of various metrics of phylogenetic diversity. All listed sequences are publicly available in the BOLD repository (http://www.boldsystems.org/). The table contains the ID number in the BOLD repository, the respective sample ID, the BIN (Barcode Index Number) of the taxon in BOLD, family, subfamily and genus affiliations of the moths, the taxon names as used in BOLD as well as in the PhD thesis of C. Truxa (viz. the original source of the moth sample data; http://othes.univie.ac.at/25605/), and information on the collectors and origin of the specimens from which the barcoding sequences in the BOLD repository had been obtained.

## Data Availability

All data generated or analysed during this study are included in this published article (and its additional files).

## Abbreviations

SD: Species diversity
PD: Phylogenetic diversity
FD: Functional diversity
NTI: Nearest Taxon Index
NRI: Net Relatedness Index
Rao: Rao’s Q for PD
FDiv: Functional divergence
FDis: Functional dispersion
FEve: Functional evenness
FRic: Functional richness
Sobs: Observed species richness
Chao: Chao1 estimator of extrapolated species richness
Hʹ: Shannon’s logarithmic diversity
expHʹ: Shannon’s exponential diversity
bc_expHʹ: Bias-corrected Shannon’s exponential diversity
alpha: Fisher’s alpha
Hill_N2 and Hill_Ninf: Hill diversity numbers
Simpson: Simpson’s lambda
J: Pielou’s evenness
E: Buzas-Gibson’s evenness
Margalef: Margalef’s d
