# Supplementary material for: The trinity of ecological contrasts: a case study on rich insect assemblages by means of species, functional and phylogenetic diversity measures
Source: BMC Ecol. 2020 May 10;20:29. doi: 10.1186/s12898-020-00298-3 (PMC7211340; doi:10.1186/s12898-020-00298-3)
Supplement: Supplementary file 1 — Additional file 1. Overview of supplementary measures of species diversity, evenness and phylogenetic diversity of moth communities. Means (95% confidence limits) of 12 additional measures for species and phylogenetic diversity across 28 moth assemblages in three east Austrian riverine regions, according to the flood regime of the forest stands. Panels refer to: observed specie richness (Sobs); Pielou’s evenness (J); Simpson’s lambda; Fisher’s alpha; Shannon’s logarithmic diversity (Hʹ); Shannon’s exponential diversity (expHʹ); the indexes of Margalef, Menihinck, and Brillouin; the Hill numbers N2 and Ninf; and the net relatedness index (NRI). [file 12898_2020_298_MOESM1_ESM.pdf]

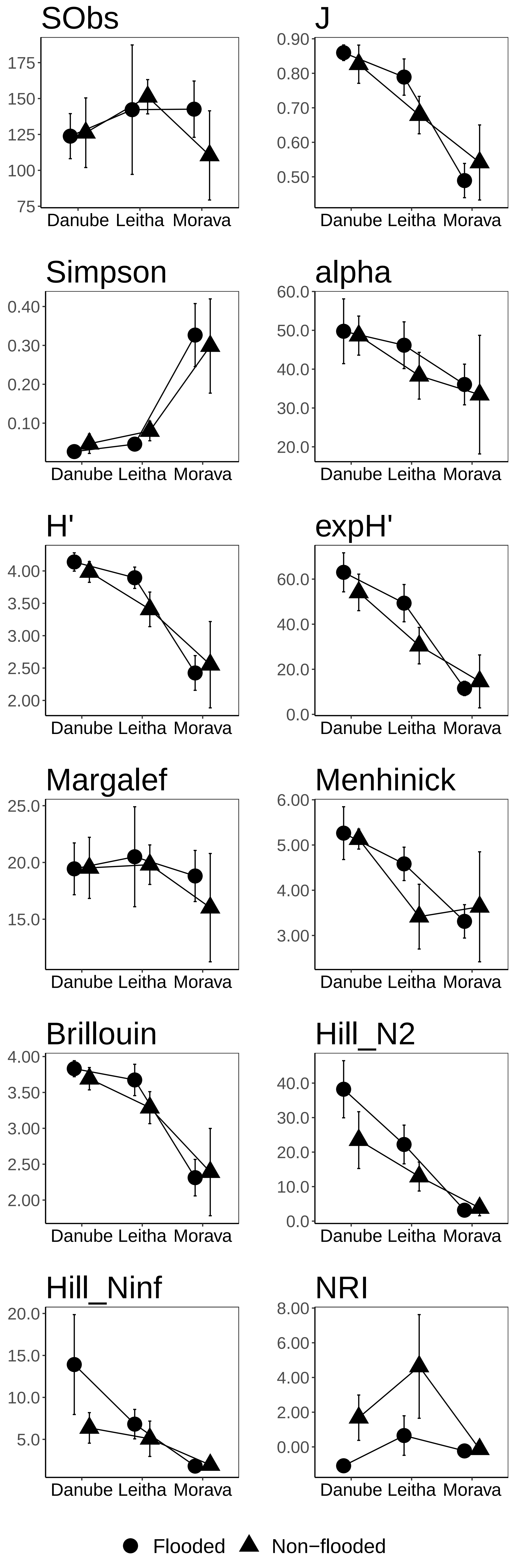

SObs
J
Simpson
alpha
H'
expH'
Margalef
Menhinick
Brillouin
Hill_N2
Hill_Ninf
NRI
Danube
Leitha
Morava
Flooded
Non−flooded
