# Supplementary figures and images for: The trinity of ecological contrasts: a case study on rich insect assemblages by means of species, functional and phylogenetic diversity measures
Source: BMC Ecol. 2020 May 10;20:29. doi: 10.1186/s12898-020-00298-3 (PMC7211340; doi:10.1186/s12898-020-00298-3)

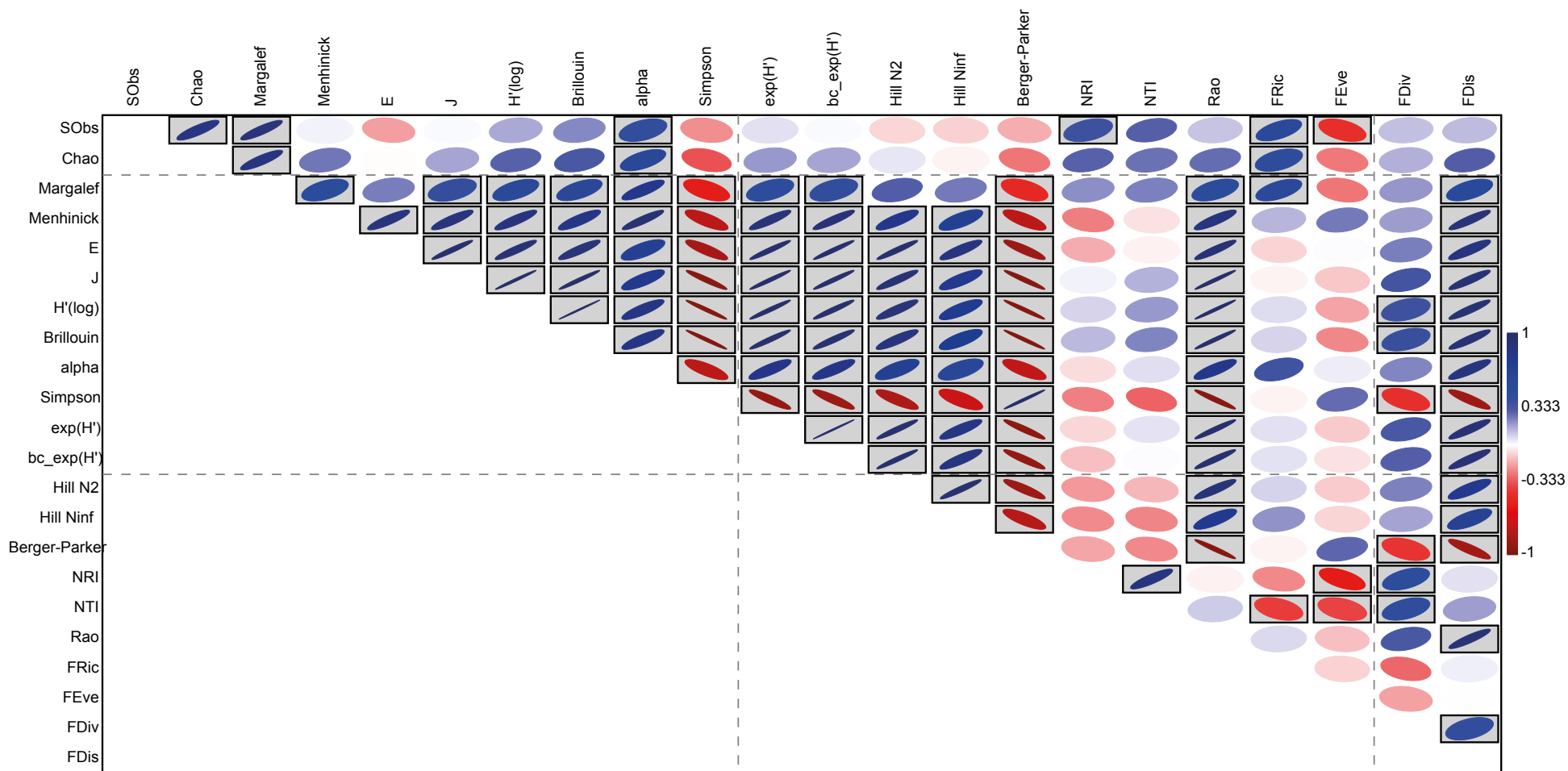

Supplement: Supplementary file 2 — Additional file 2. Correlations between 22 measures of species diversity (and evenness), functional diversity, and phylogenetic diversity. Graphical display of strength and direction of Pearson correlations between 22 measures of species, functional, and phylogenetic diversity across 28 moth assemblages. Blue: positive correlations; red: negative correlations; shaded in grey: statistically significant after sequential Bonferroni correction. More narrow and darker ellipses indicate stronger covariance. [file 12898_2020_298_MOESM2_ESM.pdf]
