# Supplementary material for: The trinity of ecological contrasts: a case study on rich insect assemblages by means of species, functional and phylogenetic diversity measures
Source: BMC Ecol. 2020 May 10;20:29. doi: 10.1186/s12898-020-00298-3 (PMC7211340; doi:10.1186/s12898-020-00298-3)
Supplement: Supplementary file 5 — Additional file 5. Additional information concerning the origin and scoring of traits enlisted in Additional file 4. Sources for species trait information on 447 moth species sampled at 28 forest sites in 3 riverine regions of lowland easternmost Austria, and a list of the traits and their scoring for analysis. [file 12898_2020_298_MOESM5_ESM.docx]

**Additional information concerning the origin and scoring of traits enlisted in Additional file 2**

Sources for species trait information on 447 moth species sampled at 28 forest sites in 3 riverine regions of lowland easternmost Austria, and a list of the traits and their scoring for analysis.

Faunal monographs:

Ahola, M., & Silvonen, K. (2005‒2011). Larvae of northern European Noctuidae. Volumes 1‒3. Kuva Seppälä, Vaasa.

Ebert, G. (ed.) (1994‒2005). Die Schmetterlinge Baden-Württembergs*.* Volumes 3‒10. E. Ulmer, Stuttgart.

Goater, B., Nuss, M., & Speidel, W. (2005). Microlepidoptera of Europe, volume 4: Pyraloidea (Crambidae: Acentropinae, Evergestinae, Heliothelinae, Schoenobiinae, Scopariinae). Apollo Books, Stenstrup.

Hausmann, A. (ed.) (2001‒2015). The geometrid moths of Europe (Volumes 1‒5). Apollo Books, Stenstrup.

Lepidopterologen-Arbeitsgruppe (1997‒2000). Schmetterlinge und ihre Lebensräume. Arten, Gefährdung, Schutz. Volumes 2 & 3. Pro Natura, Egg.

Palm, E. (1986). Nordeuropas Pyralider: med særligt henblik på den danske fauna (Lepidoptera: Pyralidae). Fauna Bøger, Copenhagen.

Rákosy, L. (1996). Die Noctuiden Rumäniens (Lepidoptera: Noctuidae). Oberösterreichisches Landesmuseum, Linz.

Riley, A., & Prior, G. (2003). British and Irish pug moths – a guide to their identification and biology. Harley, Colchester.

Skou, P. (1984). Nordens målere: håndbog over de danske og fennoskandiske arter af Drepanidae og Geometridae (Lepidoptera). Apollo Books, Svendborg.

Skou, P. (1991). Nordens ugler: håndbog over de i Danmark, Norge, Sverige, Finland og Island forekommende arter af Herminiidae og Noctuidae (Lepidoptera). Apollo Books, Stenstrup.

Slamka, F. (2008‒2013). Pyraloidea (Lepidoptera) of Central Europe: identification, distribution, habitat, biology. Volumes 1‒3. F. Slamka, Bratislava.

Sterling, P., & Parsons, M. (2012). Field guide to the micro-moths of Great Britain and Ireland. British Wildlife Publishing, Gillingham.

Trusted web-sites:

<http://www.lepiforum.de/>

<https://ukmoths.org.uk/>

<http://www.pyrgus.de/>

<http://www.euroleps.ch/>

Definition and scoring of traits:

Diurnal_activity: 0 – strictly nocturnal; 1 – adults at least partially flying during daytime

EU_territories: number of European territories (sensu Karsholt & Razowski 1996) from which a species is known. Reference: Karsholt, O., & Razowski, J. (eds.) (1996): The Lepidoptera of Europe: a distributional checklist. Apollo Books, Stenstrup.

Range_Latitude: difference (in degrees latitude) between northernmost and southernmost records within Europe, taken from the GBIF repository: <https://www.gbif.org/>.

Wingspan: geometric mean of minimum and maximum wingspan, extracted from literature. If relevant, values for both sexes and multiple generations were aggregated.

Female_flightless: 0 – females with wings well developed; 1 – females with reduced wings, unable to fly.

Voltinism: 0.5 – individuals usually take two years to complete development; 1 – one generation per year; 1.5 – at least a partial second generation develops in eastern Austria; 2 – two generations occur regularly in eastern Austria; 2.5 – under favourable climatic conditions a partial third generation occurs; 3 – three (or more) generations per year regularly seen in eastern Austria.

Hibernation_Larva: 0 – no hibernation in larval stage recorded; 1 – hibernation in larval stage recorded.

Hibernation_Pupa: 0 – no hibernation in pupal stage recorded; 1 – hibernation in pupal stage recorded.

Hibernation_Egg: 0 – no hibernation in egg stage recorded; 1 – hibernation in egg stage recorded.

Hibernation_Imago: 0 – no hibernation as adult recorded; 1 – hibernation in adult stage recorded.

Larvae_endophagous: 0 – larvae are external feeders throughout their life; 0.5 – larva construct closed shelters (from leaf rolls, leaves spun together or alike; viz. they are ‘semi-concealed’); or they are boring into plant tissues, but only for a fraction of their development; 1 – larvae are internal feeders inside plant organs such as stems, fruits, or roots.

Larvae_social: 0 – larvae feed solitarily; 0.5 – larvae form aggregations in earlier instars, sometimes in loose webs; 1 – larvae are highly social, in communal webs.

Pupa_in_on_ground: 0 – pupation site distinctly above ground, in higher vegetation; 1 – pupation site in or close to ground in low vegetation (<30 cm), thus prone to flood events.

Egg_larvae_arboreal: 0 – oviposition and larval feeding predominately occur in the herb layer (<50 cm), thus prone to flood events; 1 – oviposition and larval feeding predominately occur well above ground (>100cm), thus hardly threatened by flood events.

Larval_host-plant_families: number of host families recorded in evaluated sources. Species known to feed on plant species in just one genus are scored as 0.5.

Functional_proboscis: 0 – proboscis reduced an non-functional; 0.5 – proboscis reduced, but can be used to access at least water and very open nutrient sources; 1 – proboscis well developed.

Imago_on_non-nectar_food-sources: 0 – adults feed on flower nectar; 1 – adult moths known to regularly visit rotting fruits an related non-floral nutrient sources.

Larvae_on_dead_plant_matter: 0 – larvae feed on living plant tissues; 1 – larvae feed on withered plants or on leaf litter and alike.

Larvae_on_mosses-lichens: 0 – larvae feed on vascular plants; 1 – larvae feed on lichens, mosses, or algal biofilms.

Larvae_on_reed_aquatic: 0 – larva on terrestrial plants; 1 – larvae feed on reed (*Phragmites australis*), other reed plants (e.g. *Iris pseudacorus*) directly at the edge of water bodies, or in submersed manner on aquatic plants.

Larvae_on_herbs: 0 – larvae exclusively feed on woody plants; 0.5 – larvae mainly on woody plants, but also recorded from herbaceous plants; larvae only feed on herbaceous plants.

Larvae_on_grasses: 0 – larvae never feed on graminoid plants (Poaceae, Cyperaceae, Juncaceae); 0.5 – larval host range includes graminoid plants, but moth species is not specific to that resource; 1 – larvae exclusively known to feed on graminoid plants in nature.

Larvae_on_softwood_trees: 0 – larval host range does not include softwood trees, which are characteristic elements of floodplain forest vegetation (viz. Populus, Salix, Alnus); 0.5 – larval host range does include softwood trees; 1 – larvae exclusively feed on softwood trees in nature.

Larvae_on_lianas: 0 – larvae not known to feed on the few lianas native to Central Europe (*Clematis vitalba, Hedera helix*) which are common in floodplain forests; 0.5 – larval host range does include lianas; 1 – larvae exclusively feed on these lianas.

Larvae_on_broadleaved_trees_shrubs: 0 – caterpillars do not feed on woody deciduous plants in nature; 0.5 – host range includes broadleaved trees and shrubs; 1 – larvae only feed on trees and shrubs.

Larvae_subterraneous: 0 – larvae live above ground (except perhaps for pupation); 0.5 – larvae in uppermost litter layer, e.g. when feeding on mosses etc.; 1 – larvae spend most of their time below ground, e.g. as root herbivores.

Migratory_species: 0 – species not migratory, even though dispersive adults may show up far away from their breeding grounds; 1 – species known to perform (seasonal) long-distance migrations from southern Europe (Mediterranean) into central Europe (and back).
